# Selective Polarity Control of Metal Oxide Semiconductors for Complementary Logic Gates

**DOI:** 10.1002/advs.76809

**Published:** 2026-07-29

**Authors:** Dong Hyun Park, Seung Ho Ryu, Min Su Kim, Taek‐Mo Chung, Seong Keun Kim, In‐Hwan Baek, Keun Hyung Lee

**Affiliations:** ^1^ Department of Chemistry and Chemical Engineering Education and Research Center for Smart Energy and Materials Inha University Incheon Republic of Korea; ^2^ Electronic and Hybrid Materials Research Center Korea Institute of Science and Technology Seoul Republic of Korea; ^3^ KU‐KIST Graduate School of Converging Science and Technology Korea University Seoul Republic of Korea; ^4^ Division of Advanced Materials Korea Research Institute of Chemical Technology Daejeon Republic of Korea

**Keywords:** complementary logic, electrochemical doping, electrolyte‐gated transistor, metal oxide semiconductor, polarity conversion

## Abstract

Realizing tunable charge carrier polarity is essential for programmable complementary logic circuits. While two‐dimensional (2D) semiconductors have demonstrated polarity controllability through Schottky barrier modulation or contact engineering, their high interfacial sensitivity limits scalability and integration. Here, we present a robust polarity‐conversion strategy for tin oxide (SnO) semiconductors based on electrolyte‐gated transistors (EGTs). In situ electrochemical doping enables the conversion of p‐type SnO to n‐type SnO_2_ via Sn^2+^ to Sn^4+^ oxidation. Both p‐ and n‐type EGTs exhibit outstanding electrical characteristics, including low‐voltage operation, high ON/OFF current ratios, sharp switching behavior, and reliable long‐term stability. Furthermore, we demonstrate the monolithic integration of complementary logic gates, including inverter, NAND, and NOR gates, through spatially selective electrochemical doping of a single SnO layer. Overall, this work provides a scalable and versatile pathway toward oxide‐based programmable complementary circuits.

## Graphical abstract

A scalable electrochemical doping strategy enables in situ p‐to‐n polarity conversion in tin oxide (SnO) thin films using electrolyte gating. Both the pristine p‐type SnO transistor and the electrochemically converted n‐type SnO_2_ transistor exhibited noticeable and stable electrical performance. Monolithic logic circuits are fabricated by spatially selective phase conversion, eliminating the need for separate n‐type transistors.

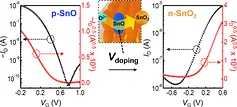

## 1 Introduction

Precise control over semiconductor polarity is a fundamental requirement for realizing programmable complementary logic circuits [1, 2, 3, 4]. Traditional doping techniques, such as ion implantation, allow accurate tuning of carrier type and concentration. However, the polarity of the resulting semiconductors is irreversibly determined. Two‐dimensional (2D) semiconductors, including transition metal dichalcogenides (TMDs) and black phosphorus (BP), have emerged as promising candidates for doping‐free polarity control owing to their ambipolar transport characteristics and atomically thin structure. Various strategies, such as Schottky barrier tuning, contact engineering, and Fermi‐level pinning, have been proposed to electrostatically modulate semiconductor polarity [1, 3, 4, 5, 6, 7, 8, 9]. For instance, BP‐based transistors have demonstrated programmable logic gates through gate‐controlled Schottky barrier tuning [3], while WSe_2_‐based circuits have enabled doping‐free logic via the selective integration of a van der Waals metal [4]. Despite these advances, the extreme surface sensitivity and fabrication complexity of the 2D materials remain significant challenges, especially for scalable, large‐area, and reliable circuit integration [10, 11].

As an alternative, oxide semiconductors offer favorable properties including chemical robustness and thermal stability, along with compatibility with low‐temperature fabrication techniques [12, 13, 14]. These benefits are further enhanced when oxide semiconductors are deposited by atomic layer deposition (ALD), which enables sub‐nanometer thickness control with exceptional conformality and uniformity across large‐area substrates [14, 15]. Such process‐level precision is indispensable for programmable logic circuits, as it ensures device‐to‐device performance uniformity that is critical for reliable and deterministic polarity conversion. Among oxide semiconductors, tin oxide (SnO) uniquely exhibits high hole mobility, attributed to a valence band maximum (VBM) derived from the hybridization of O 2p and Sn 5s orbitals, which enables efficient hole transport [16]. Accordingly, ALD‐grown SnO can serve as a promising platform for programmable logic circuits, particularly well‐suited for p‐to‐n polarity modulation.

Furthermore, recent studies show that electric‐field‐driven ionic motion in ionic liquids (ILs) or ionogels can significantly modulate the electrical and magnetic properties of functional oxides [17, 18]. Unlike conventional electrostatic gating, which merely accumulates charge carriers at the interface, electrochemical gating can drive ions such as O^2−^ or H^+^ into and out of the oxide lattice, leading to deep, often non‐volatile changes in phase, oxidation state, and conductivity [17, 18]. For example, phase transformations in various oxides, including SrTiO_3_, VO_2_, and SrCoO_x_, have been achieved through electrochemical doping, allowing metal‐insulator transition, magnetic ordering, and even superconductivity [19, 20, 21, 22, 23, 24, 25, 26].

In this study, we demonstrate a facile polarity‐control strategy based on electrochemical phase conversion in the SnO semiconductor using an electrolyte‐gated transistor (EGT). By leveraging strong electric fields at the ionogel/SnO interface, we induce oxidation of Sn^2+^ to Sn^4+^, enabling in situ conversion of p‐type SnO to n‐type SnO_2_. Both p‐ and n‐type EGTs exhibit excellent electrical characteristics, including high mobility, large ON/OFF current ratios, and sharp subthreshold swings, all under sub‐1 V operation. Building on this controllable phase conversion, monolithic integration of complementary logic gates, including inverters, NAND, and NOR, within a single SnO semiconductor was successfully demonstrated through spatially selective electrochemical doping. This process eliminates the need for separate n‐type semiconductors and provides a simplified, scalable pathway toward oxide‐based programmable complementary logic circuits. These findings highlight electrochemical polarity conversion in oxide semiconductors as a promising strategy for realizing complementary thin‐film logic circuits.

## 2 Results and Discussion

Figure 1 illustrates polarity conversion of SnO from p‐type to n‐type via electrochemical doping in an EGT. Initially, the p‐type SnO channel was deposited via ALD (Figure 1a), and source/drain electrodes were defined on the SnO by photolithography to form a top‐contact architecture. Subsequently, an ionogel gate electrolyte and a single‐walled carbon nanotube (SWCNT)‐based gate electrode were sequentially laminated to complete the top‐contact/top‐gate EGT structure. The ionogel consists of poly(vinylidene fluoride‐*co*‐hexafluoropropylene) (PVDF‐HFP) network polymer and 1‐ethyl‐3‐methylimidazolium bis(trifluoromethylsulfonyl)imide ([EMI][TFSI]) (Figure S1). The large areal capacitance (>1 *µ*F cm^−2^) of the SWCNT‐gate/ionogel interface, originating from electric double layer (EDL) formation, enables sufficient hole or electron accumulation at the semiconductors [27].

**FIGURE 1:**
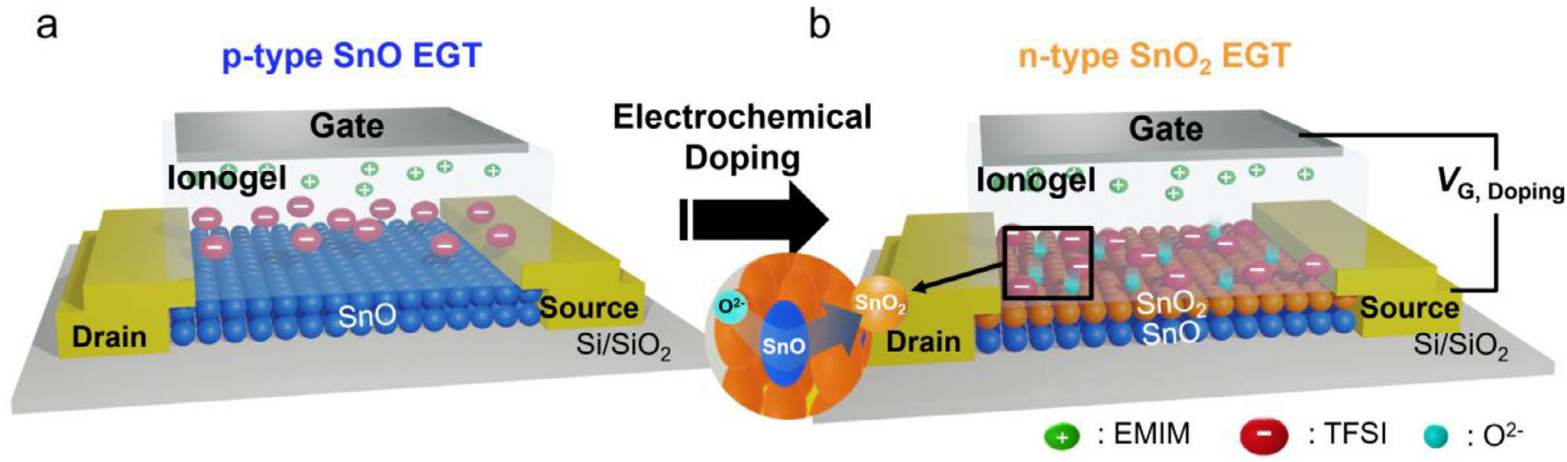
Schematic of the polarity control process in an electrolyte‐gated SnO semiconductor. (a) Device configuration of the p‐type SnO EGT before electrochemical doping. (b) Formation of the n‐type SnO_2_ EGT after electrochemical doping by applying gate potential (*V*_G, Doping_ = −1.5 V).

To convert the as‐deposited p‐type SnO semiconductor into n‐type SnO_2_, electrochemical doping was conducted. Upon applying a gate bias *V*_G, Doping_ = −1.5 V, the nanometer‐thin EDL at the ionogel/SnO interface generates an ultrahigh electric field on the order of MV cm^−1^. Such intense interfacial electric fields can exceed the electrochemical threshold for the electrolysis of trace water present in the IL, generating O^2−^ ions that oxidize Sn^2+^ to Sn^4+^, thereby leading to the formation of oxygen‐rich SnO_2_ at the ionogel interface [20, 28, 29, 30]. As a result, an in situ transformation from p‐type SnO to n‐type SnO_2_ was achieved within a single material, as schematically shown in Figure 1b. To the best of our knowledge, such oxidation‐state‐driven polarity conversion has not previously been demonstrated in oxide thin‐film transistors. Note that the pristine p‐type SnO film remained chemically stable during field‐effect gating with a 100 nm‐thick SiO_2_ dielectric under ambient conditions (Figure S2).

The associated chemical changes were characterized by X‐ray photoelectron spectroscopy (XPS) on both the pristine p‐type SnO (p‐SnO) and the n‐type SnO_2_ (n‐SnO_2_) films. Figure S3a reveals that the Sn 3d_5/2_ peak of pristine SnO appears at 486.1 eV, corresponding to the Sn^2+^ oxidation state [31]. The pristine SnO also exhibits the Sn^4+^ peak at 487 eV, which is attributed to partial surface oxidation caused by its metastable nature, consistent with previously reported observations [16]. After electrochemical doping at *V*_G_ = −1.5 V, the Sn^4+^ peak at 487 eV becomes pronounced, indicating the formation of the Sn^4+^ state (Figure S3b) [32]. The extent of polarity conversion was quantitatively assessed by comparing the Sn^4+^/Sn^2+^ peak area ratio before and after electrochemical doping (Figure S4). The average ratio increased from 0.47 to 1.87, clearly indicating the formation of n‐type SnO_2_. Note that the observed Sn^2+^ signal in the SnO_2_ likely originates from the underlying pristine SnO, given the XPS probing depth (∼8 nm).

To evaluate the structural evolution accompanying the electrochemical Sn^4+^ conversion, high‐resolution transmission electron microscopy (HR‐TEM) was performed across the entire SnO thin‐film, including the electrolyte/semiconductor interface. Notably, the cross‐sectional HR‐TEM image in Figure 2a reveals a strong contrast between the SnO near the substrate and the n‐type SnO_2_ adjacent to the electrolyte interface. As shown in the magnified images of regions 1 and 2, the SnO at the electrolyte interface transforms into a new crystalline phase, with lattice d‐spacings of 3.7 and 2.4 Å corresponding to the (110) and (200) planes of orthorhombic SnO_2_ (ICSD 62199), respectively. This structural conversion from SnO (ICSD 26597, Figure S5) to SnO_2_ is consistent with the increased Sn^4+^ peak intensity observed in the XPS spectra, further supporting the successful phase transformation induced by electrolyte gating.

**FIGURE 2:**
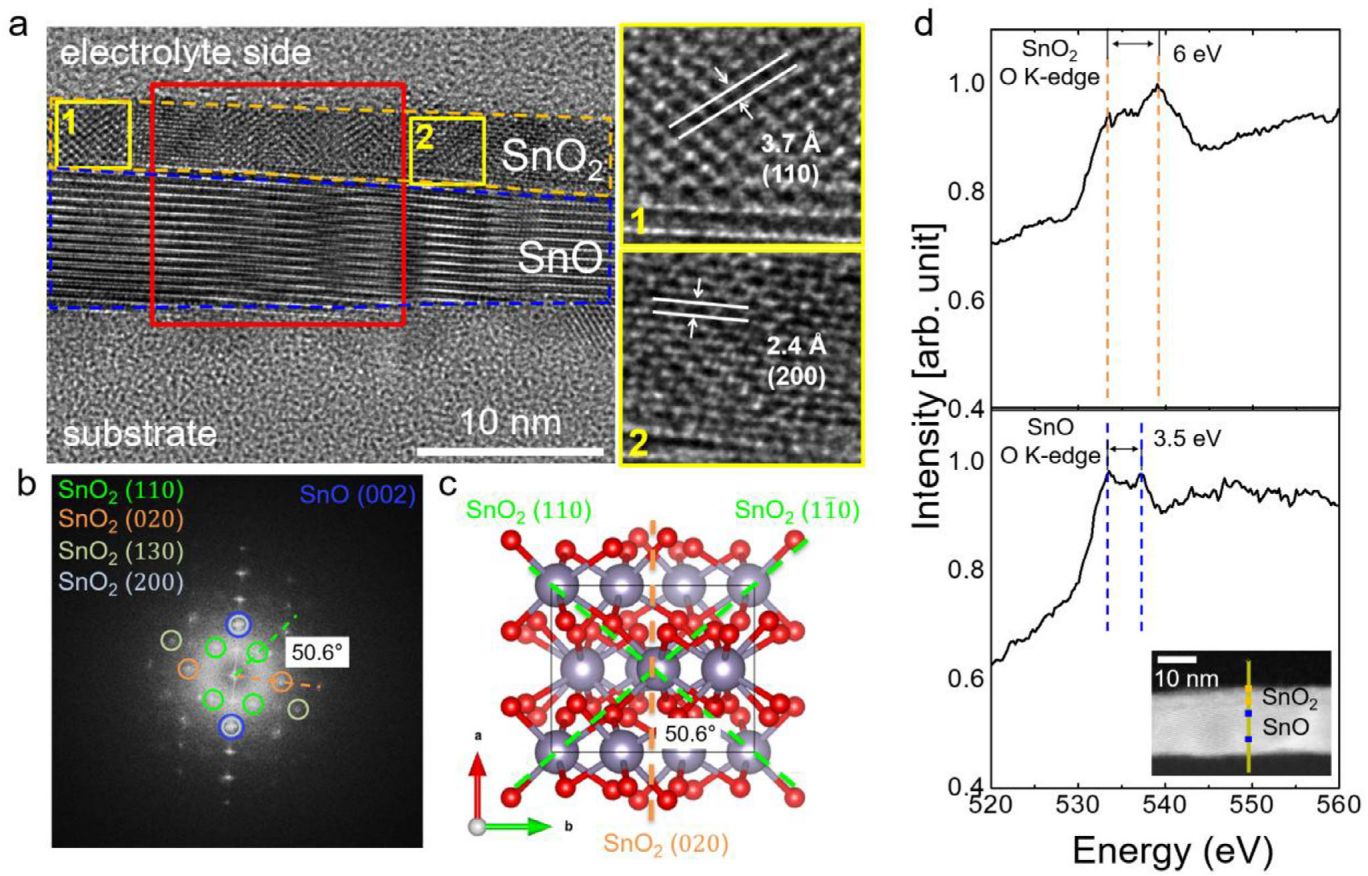
Structural analysis of the SnO_2_. (a) Cross‐sectional HR‐TEM image of the SnO_2_ thin‐film. (b) Corresponding Fast Fourier Transform (FFT) of the red‐boxed region shown in a), including both SnO_2_ and SnO phases. (c) Schematic of the orthorhombic SnO_2_ crystal structure (ICSD 62199). (d) EELS spectra of the oxygen K‐edge for SnO_2_ (top) and SnO (bottom). The inset in d) indicates the locations where the EELS spectra were acquired.

A more detailed structural assessment was carried out through Fast Fourier Transform (FFT) analysis of the red‐boxed region in Figure 2a, and the result is presented in Figure 2b. Except for the pristine SnO (002) plane near the substrate, all diffraction spots were indexed to orthorhombic SnO_2_. Notably, a clear alignment between the SnO (002) and SnO_2_ (200) planes was observed, affirming a specific crystallographic orientation between the two phases. This coherent crystallographic relationship suggests that the phase conversion from SnO to SnO_2_ proceeds via oxygen incorporation under gate bias, without requiring significant diffusion of the heavier Sn atoms. The new SnO_2_ phase exhibits an interplanar angle of 50.6° between the (110) and (020) planes, matching the calculated value for orthorhombic SnO_2_ (ICSD 62199), as shown in Figure 2c.

To prove the depth‐dependent phase conversion, oxygen K‐edge electron energy loss spectroscopy (EELS) spectra were acquired from both the surface and interior of the film (Figure 2d). The oxygen K‐edge provides a sensitive probe of the oxidation state of metal ions, as it reflects subtle variations in the coordination environment of oxygen. Near the ionogel interface, a distinct peak appears ∼6 eV above the first oxygen peak (calibrated at 532 eV), matching the spectral fingerprint of SnO_2_. In contrast, the spectrum from the interior region shows a narrower separation of 3.5 eV, indicative of SnO [33]. This spatially resolved feature provides direct chemical evidence of localized oxidation near the ionogel interface, in agreement with the HR‐TEM observations.

Figure 3a,d present representative transfer curves of the p‐type SnO (p‐SnO) and n‐type SnO_2_ (n‐SnO_2_) EGTs, measured at fixed drain voltages (*V*_D_) of −0.4 and +0.4 V, respectively. The p‐SnO turns on with decreasing gate voltage (*V*_G_), indicating hole (p‐type) transport. In contrast, the n‐SnO_2_, obtained via electrochemical doping of p‐SnO, exhibits conventional n‐type characteristics, as evidenced by the increase in drain current (*I*_D_) with increasing *V*_G_, confirming the successful polarity conversion from p‐SnO to n‐SnO_2_. The linear correlation between the square root of *I*_D_ and *V*_G_ shows stable saturation‐mode operation in both p‐ and n‐type channels (red curves). The p‐SnO EGT shows a maximum *I*_D_ of 1.1 µA at *V*_G_ = −0.2 V, whereas the n‐SnO_2_ EGT exhibits a significantly higher saturation current of 10.6 µA at *V*_G_ = 0.6 V. Accordingly n‐SnO_2_ possesses a higher saturation mobility of 1.17 cm^2^ V^−1^ s^−1^ compared to that of p‐SnO (0.17 cm^2^ V^−1^ s^−1^). The ON/OFF current ratios of the p‐SnO and n‐SnO_2_ EGTs were 1.38 × 10^4^ and 1.03 × 10^3^, respectively. The subthreshold swing values of the p‐ and n‐type EGTs were 0.163 and 0.277 V/dec, respectively [34, 35, 36, 37]. Furthermore, transient measurements revealed turn‐on (τ_ON_)/turn‐off (τ_OFF_) time constants of 28.6/7.7 ms for p‐SnO and 88.3/28.5 ms for n‐SnO_2_, respectively (Figure S6). Device parameters including hole and electron mobilities, threshold voltage, subthreshold swing, and ON/OFF current ratios for p‐SnO and n‐SnO_2_ EGTs are summarized in Table S1. Figure S7 illustrates the expected carrier transport pathways in p‐SnO and n‐SnO_2_ EGTs. While hole transport in the pristine device occurs in the p‐type SnO channel, electron transport in the converted device is expected to be dominated by the electrochemically formed SnO_2_ surface region adjacent to the ionogel, although the underlying SnO layer may still contribute to the total channel current.

**FIGURE 3:**
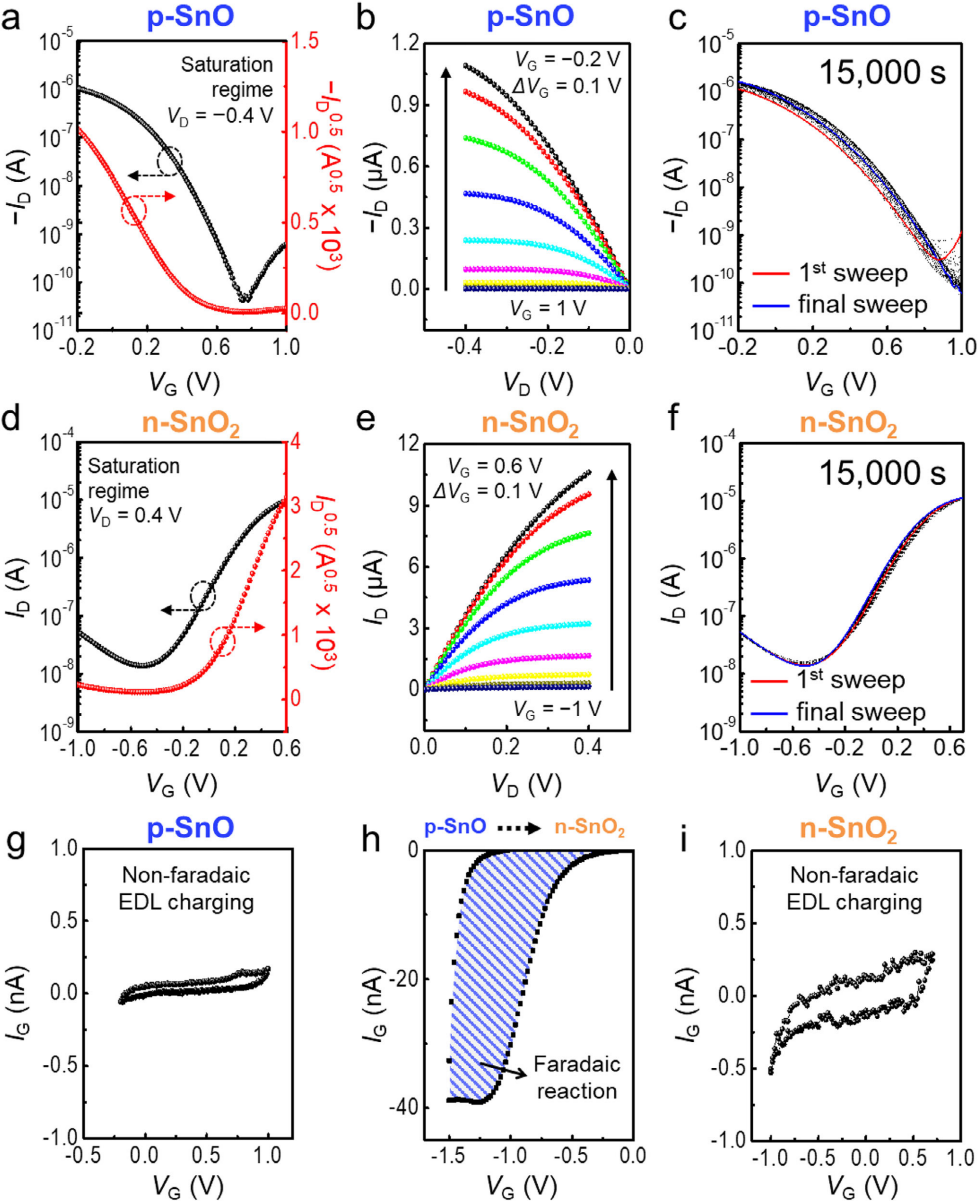
Electrical characteristics of p‐SnO and n‐SnO_2_ EGTs. Electrical performance of the p‐SnO EGT: (a) transfer characteristics (*I*_D_ *−V*_G_) in the saturation regime; (b) output characteristics (*I*_D_ *−V*_D_); and (c) operational stability over 15 000 s under repeated measurements. Corresponding data for the n‐SnO_2_ EGT, including (d) transfer; (e) output; and (f) operational stability characteristics. Gate current characteristics (*I*_G_ *−V*_G_) of (g) the pristine p‐SnO EGT; (h) during electrochemical doping; and (i) after conversion to n‐SnO_2_.

Representative output curves for p‐SnO and n‐SnO_2_ EGTs, shown in Figure 3b,e, respectively, exhibit linear increases in *I*_D_ at low *V*_D_, followed by current saturation at higher *V*_D_. Repetitive *I*_D_ *−V*_G_ measurements of the p‐SnO and n‐SnO_2_ EGTs at *V*_D_ = −0.4 and +0.4 V, respectively, demonstrate stable operation without noticeable channel current degradation or threshold voltage drift over 15 000 s (Figure 3c,f). The p‐SnO transfer curves exhibit somewhat greater variation than those of the n‐SnO_2_ devices. However, the observed variation is unlikely to originate from the conversion of SnO into SnO_2_ during normal transistor operation. If gradual SnO‐to‐SnO_2_ conversion was occurring, the p‐type transistor characteristics would deteriorate. In contrast, Figure S8 shows a slight increase in ON current and a decrease in OFF current with repeated gating cycles. This behavior is attributed to the stabilization of the SnO/ionogel interface during repeated electrolyte‐gating operations. Notably, electrochemical conversion to n‐type SnO_2_ requires a substantially larger gate voltage (−1.5 V) than the normal p‐type operating voltage (−0.2 V). These results confirm a reliable conversion of p‐type to n‐type characteristics via electrochemical doping.

The gate currents (or *I*_G_ *−V*_G_ curves) enable distinction between non‐faradaic EDL formation and faradaic redox reactions at the semiconductor/ionogel interface, analogous to cyclic voltammetry [38, 39]. Both p‐SnO and n‐SnO_2_ EGTs exhibit quasi‐rectangular *I*_G_ *−V*_G_ curves, with capacitive currents of ∼0.2 and ∼0.5 nA, respectively, indicating non‐faradaic EDL charging (Figure 3g,i). Accordingly, the charge density values extracted from the gate currents were relatively small for both p‐SnO (0.33 × 10^14^ cm^−2^) and n‐SnO_2_ (0.54 × 10^14^ cm*^−^* ^2^). In contrast, as shown in Figure 3h, when a doping gate voltage (*V*_G, Doping_) of −1.5 V was applied to p‐SnO, a pronounced gate current of ∼40 nA was recorded, nearly 200 times higher than the capacitive current (∼0.2 nA) of the p‐SnO. This increase in gate current is attributed to the faradaic oxidation of Sn^2+^ to Sn^4+^. The resulting charge density associated with the faradaic reaction reached 9.01 × 10^15^ cm^−2^, significantly higher than that of the p‐SnO. Notably, this value exceeds the typical upper limit of charge density achievable by electrostatic gating or conventional electrolyte gating (∼10^14^ cm^−2^), further confirming the presence of electrochemical reactions beyond pure capacitive charge accumulation [40, 41, 42]. Following the completion of the redox reaction converting p‐SnO to n‐SnO_2_, the gate current of the n‐SnO_2_ EGT returns to a quasi‐rectangular shape, again indicative of non‐faradaic EDL charging.

To further validate EDL‐based capacitive carrier accumulation before and after phase conversion, gate voltage sweep measurements were conducted at varying scan rates (5, 17, 40, 100, and 200 mV s^−1^) on both the p‐SnO EGT (Figure S9a) and the n‐SnO_2_ EGT (Figure S9b) [43]. In both cases, the *I*_G_ *–V*_G_ curves exhibit symmetric, quasi‐rectangular profiles that scale proportionally with scan rate, confirming non‐faradaic EDL charging as the dominant mechanism.

As summarized in Table S2, previously reported polarity‐control approaches have been demonstrated predominantly in 2D semiconductors [1, 2, 3, 5, 6, 7, 8, 9, 44, 45, 46, 47]. In contrast, the present work demonstrates electrochemical polarity conversion in an oxide semiconductor through a controllable SnO‐to‐SnO_2_ phase transformation, enabling the realization of both p‐type and n‐type transistors from a single ALD‐grown semiconductor layer via a post‐fabrication process. Moreover, because oxide semiconductors are compatible with established thin‐film deposition techniques, including low‐temperature ALD using environmentally benign oxidants such as H_2_O, the proposed post‐fabrication polarity‐conversion strategy offers a practical pathway toward scalable, monolithic complementary oxide electronics. Comparison with previously reported oxide‐based EGTs [48, 49, 50, 51, 52, 53, 54, 55, 56, 57, 58, 59, 60] (Table S3) further shows that the electrical characteristics of the present devices fall within the range reported for oxide EGTs. While state‐of‐the‐art n‐type oxide EGTs based on ZnO and indium gallium zinc oxide (IGZO) can exhibit higher mobilities and superior switching performance, reports of p‐type oxide EGTs remain limited. More importantly, the present approach uniquely enables complementary operation from a single oxide semiconductor through electrochemical phase conversion, providing a facile route toward monolithically integrated complementary oxide logic circuits.

To confirm the universality of polarity conversion using EGTs, different ILs including [EMI] tetrafluoroborate ([BF_4_]) and butyl trimethyl ammonium ([BTMA]) [TFSI] were investigated. Transfer curves in Figure S10 show that polarity conversion from p‐SnO to n‐SnO_2_ was successful for both ILs, containing different cation‐anion pairs. In contrast, when using 0.1 m NaCl aqueous electrolyte as the gate electrolyte, we failed to induce polarity conversion due to the narrow electrochemical window of water, where electrolysis occurs before SnO oxidation (Figure S11). Linear sweep voltammetry (LSV) curves in Figure S12 indicate that [EMI][TFSI] is stable without side reactions up to ∼3 V [20]. In contrast, the NaCl aqueous electrolyte undergoes electrolysis reaction at ∼1.2 V. In the aqueous electrolyte, water electrolysis is initiated before SnO oxidation because of the narrow electrochemical stability window. Consequently, the high interfacial electric field necessary to drive the SnO‐to‐SnO_2_ phase conversion cannot be sustained. In contrast, ionic liquids provide a wide electrochemical window while containing trace amounts of water, enabling both the generation of reactive oxygen species and the application of the high interfacial electric fields necessary for electrochemical SnO‐to‐SnO_2_ conversion. These results suggest that conventional aqueous electrolytes are unsuitable for gate‐induced polarity conversion, as they cannot supply sufficient voltage to drive the conversion of SnO to SnO_2_. Taken together, these findings highlight the pivotal role of ionogel electrolytes, particularly those inherently containing trace amounts of water (Table S4), in facilitating effective phase transition of SnO EGTs. Their ability to form robust EDLs and operate across wide voltage windows is essential for enabling the universal applicability of electrochemical phase control in SnO semiconductors.

It is noteworthy that SnO is a p‐type oxide semiconductor with excellent electronic properties and operational stability. Although n‐type oxide semiconductors such as ZnO and IGZO have been widely employed for electrolyte‐gated applications, stable p‐type oxide semiconductors remain scarce. Since p‐type electrolyte‐gated semiconductors are essential for complementary circuit applications, SnO represents a promising candidate for complementary oxide electronics. Compared with organic electrolyte‐gated semiconductors, Sn oxides are less susceptible to swelling and structural disorder during electrolyte gating, resulting in improved device stability and robustness.

Using electrochemical polarity conversion, we realized a monolithic complementary inverter entirely based on a single SnO semiconductor. As shown in Figure 4a, the inverter was initially constructed by connecting two p‐type SnO EGTs in series, each laminated with the ionogel dielectric and SWCNT gate electrode. Selective polarity conversion was subsequently performed on the pull‐down transistor, converting it into n‐type SnO_2_. This in situ transformation eliminated the need for separate n‐type material processing. The corresponding equivalent circuit (Figure 4b) shows the inverter configuration, in which the gate terminals of the p‐ and n‐type EGTs are connected to serve as the input node. Figure 4c presents the voltage transfer characteristics of the monolithic complementary inverter at three different supply voltages (*V*_DD_). A sharp voltage transition and full rail‐to‐rail output swing were observed at a narrow input voltage (*V*_In_) of 0.1 V, with a high voltage gain (*dV*_Out_/*dV*_In_) of 19.3 at *V*_DD_ = 0.9 V (Figure 4d). More importantly, these results demonstrate that selectively converted SnO/SnO_2_ devices can function as complementary circuit building blocks. By enabling both p‐type and n‐type operation from the same oxide material through electrochemical polarity conversion, this approach eliminates the need for heterogeneous integration of separate semiconductor materials and offers a viable route toward complementary oxide logic circuits.

**FIGURE 4:**
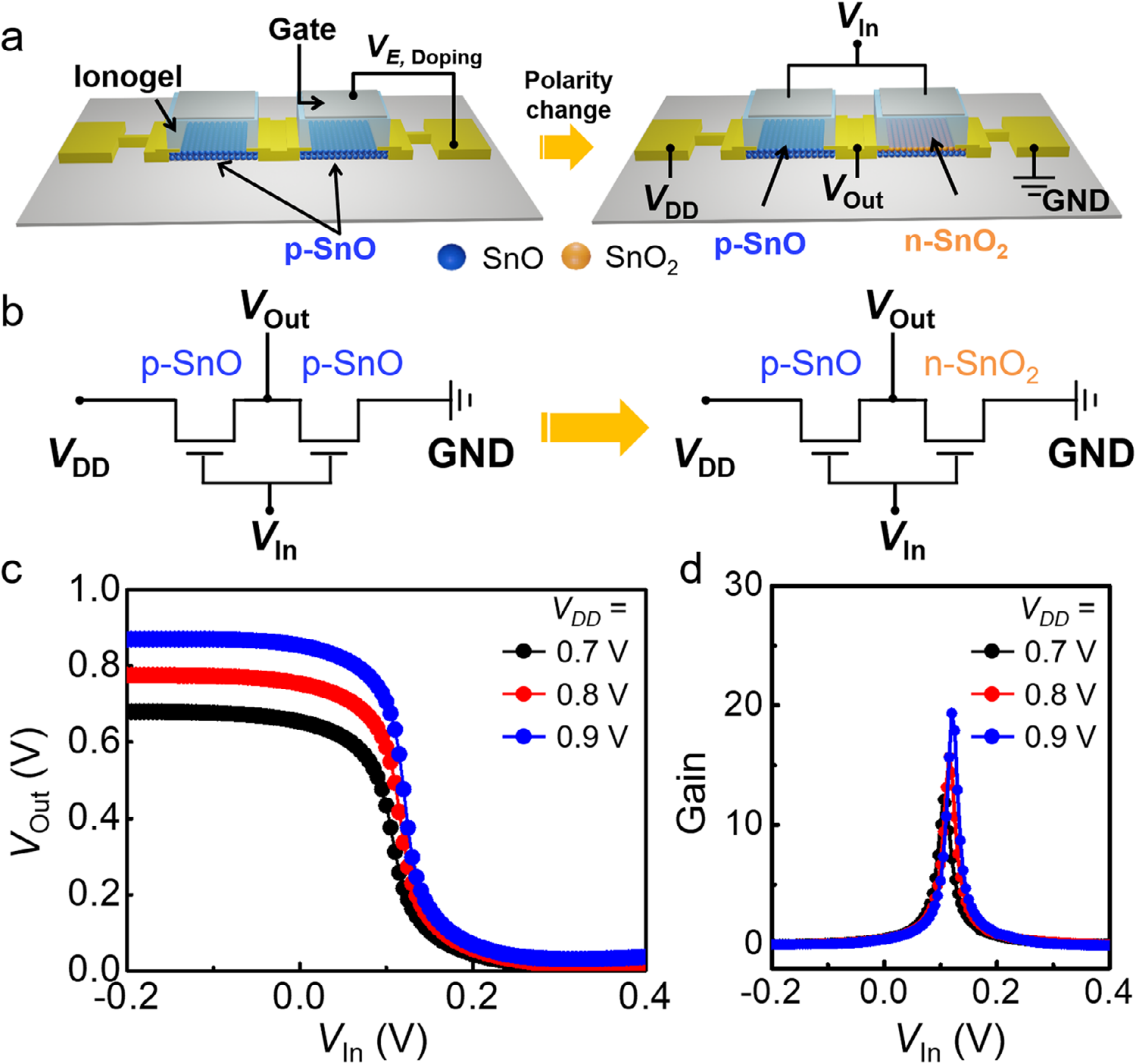
Monolithic complementary inverter enabled by polarity conversion. (a) Schematic of inverter fabrication using spatially selective polarity conversion of SnO transistors. (b) Corresponding equivalent circuits before (left) and after (right) polarity conversion. (c) Voltage transfer characteristics and (d) corresponding voltage gain of the inverter as a function of input voltage.

Selective electrochemical polarity conversion was employed to fabricate more complex complementary logic gates, specifically NAND and NOR, which are fundamental building blocks of digital logic. Figure 5a shows the initial configuration comprising four p‐SnO EGTs, two arranged in series and two in parallel, along with the corresponding truth table for the NAND and NOR gates. To form a NAND gate, the series‐connected p‐SnO EGTs were selectively converted to n‐SnO_2_, while converting the parallel‐connected p‐SnO EGTs yielded a NOR gate. Optical images of the NAND and NOR gates are shown in Figure 5b,c, respectively. To evaluate logic functionality, input signals *V*_A_ and *V*_B_ were applied in various logic state combinations, with *V*_DD_ set to 0.7 V. An input logic state of “0” was defined when both *V*_A_ and *V*_B_ were set to −0.2 V, while a logic state of “1” corresponded to an input voltage of 0.4 V. Figure 5d shows the time‐dependent voltage step inputs for each signal (*V*_A_, *V*_B_). The NAND gate produced an output voltage of *V*_DD_ (0.7 V, logic “1”) for input combinations (0,0), (1,0), and (0,1), while an output of *V*_OUT_ = 0 V (logic “0”) was observed only for the (1,1) input (Figure 5e). The NOR gate successfully demonstrated its characteristic behavior by outputting *V*_DD_ (logic “1”) only for the (0,0) input, while all other input combinations resulted in a logic “0” output (Figure 5f). These results confirm that complex logic functions can be reliably implemented through selective electrochemical polarity control within a single‐material system. Overall, our approach offers a versatile and scalable platform for the monolithic integration of complementary logic circuits using oxide semiconductors.

**FIGURE 5:**
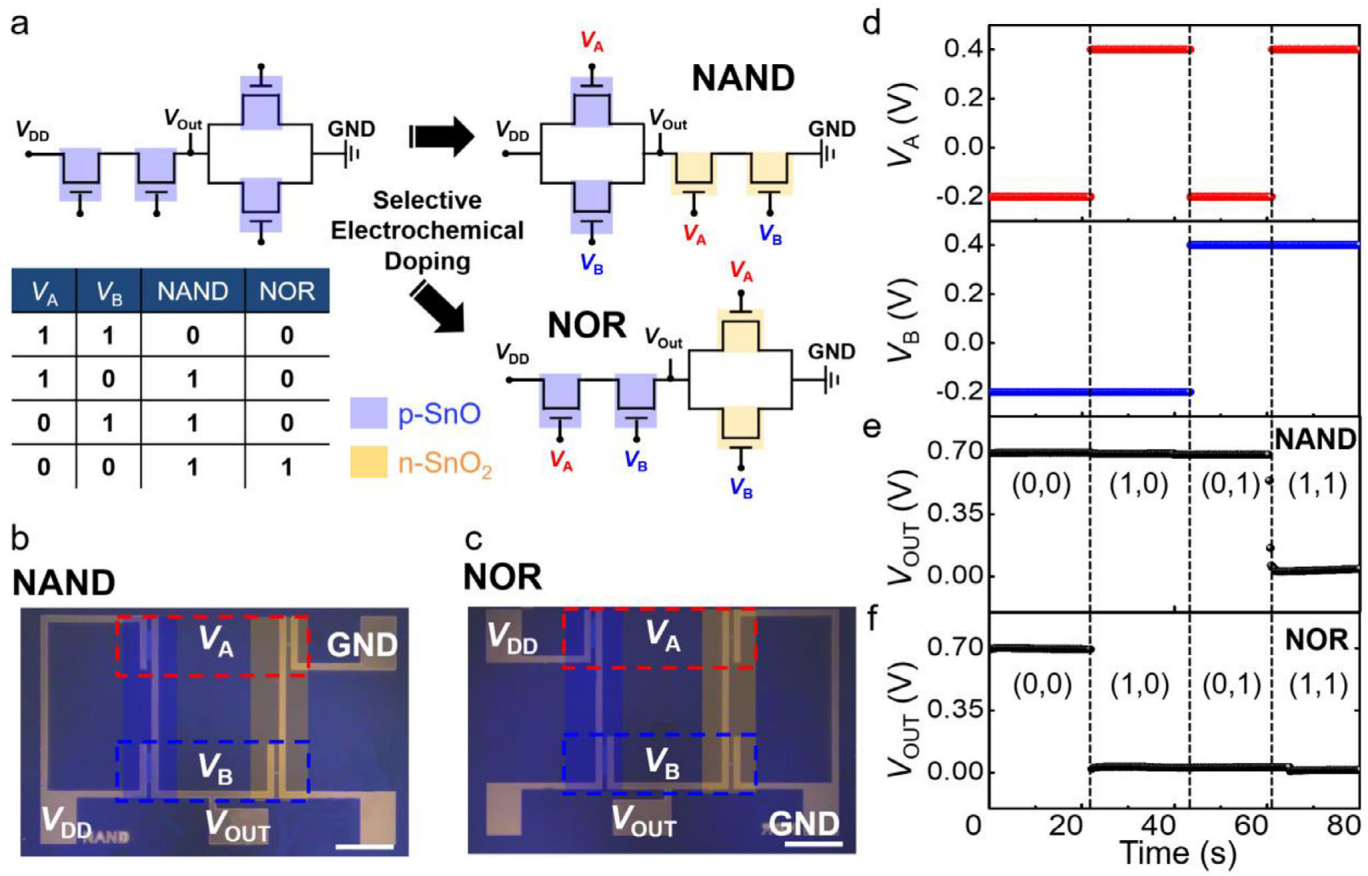
Selective polarity conversion enables NAND and NOR gates. (a) Schematic of complementary NAND and NOR gate fabrication via selective polarity control, along with the corresponding truth table. Four p‐type SnO EGTs were initially prepared (top left). Selective conversion of the series‐connected EGTs forms a NAND gate (top right), while conversion of the parallel‐connected EGTs results in a NOR gate (bottom right). Optical images of the fabricated (b) NAND and (c) NOR gates. (d) Input signals with four different logic state combinations. Output voltage responses of the (e) NAND and (f) NOR gates.

## 3 Conclusion

In this study, we demonstrated a polarity control strategy for p‐type SnO, enabling its in situ transformation into n‐type SnO_2_ via electrochemical doping under strong electric fields induced by ionogel gating. Gate current analyses revealed that faradaic reactions drive the polarity transition. Both p‐SnO and n‐SnO_2_ EGTs exhibited stable operation for over 15 000 s, with high mobility, large ON/OFF current ratios, and sharp switching behavior, without voltage or current drift. Leveraging selective electrochemical doping, we successfully fabricated complementary logic circuits, including inverters, NAND, and NOR gates, using a single SnO material. Logic gates exhibited full rail‐to‐rail output swing and correct logic‐level responses across all input combinations. Overall, our approach provides a simplified, scalable, and cost‐effective strategy for implementing complementary logic in thin‐film oxide electronics, eliminating the need for separate n‐type materials.

## 4 Experimental Section

### 4.1 Materials

[EMI][TFSI] (99.5%) was purchased from Solvionic Inc. P(VDF‐HFP), acetone (>99.9%), dimethylformamide (DMF), [BTMA][TFSI], and [EMI][BF_4_] were obtained from Sigma–Aldrich. The bis(1‐dimethylamino‐2‐methyl‐2‐propoxy)tin(II) (Sn(dmamp)_2_) precursor for SnO semiconductor was synthesized at the Korea Research Institute of Chemical Technology. Single‐walled carbon nanotubes (SWCNTs, >99%) for the gate electrode were obtained from Meijo Nano Carbon, and conductive carbon black (Super P) was purchased from Timcal. Polydimethylsiloxane (PDMS, Sylgard 184) was purchased from Dow Corning.

### 4.2 Preparation of Ionogel and Gate Electrode

Ionogel gate dielectrics were prepared by dissolving P(VDF‐HFP) in acetone at 50°C for 2 h, followed by the addition of ionic liquids and further mixing for 2 h. The weight ratio of P(VDF‐HFP): ionic liquid: acetone was fixed at 1:4:7. The resulting solution was drop‐cast onto a glass Petri dish and dried overnight to remove the residual solvent. The dried ionogel film was subsequently cut and laminated onto devices as the gate dielectric layer. SWCNT gate electrodes were fabricated by dispersing SWCNTs, Super P, and P(VDF‐HFP) in a weight ratio of 8:1:1 in DMF using bath sonication for 2 h. The dispersion was drop‐cast onto a PDMS mold and dried at 50°C. This coating step was repeated to obtain a three‐layer SWCNT film, ensuring sufficient gate capacitance.

### 4.3 SnO and Ionic Liquid Characterizations

XPS was conducted using a Nexsa‐G2 system to analyze the chemical states of p‐SnO and n‐SnO_2_. Samples were sequentially cleaned with acetone and isopropyl alcohol, followed by nitrogen drying. The C 1s peak at 284.8 eV was used for binding energy calibration. HR‐TEM and EELS measurements were performed using a Titan microscope (FEI Company). Samples were cleaned using the same procedure as for XPS analysis, and then focused ion beam (FIB) milling was performed to prepare the TEM specimens. LSV measurements of [EMI][TFSI] and 0.1 m NaCl aqueous solution were performed using a potentiostat (VersaSTAT 4). A metal‐insulator‐semiconductor‐metal (MISM) device was fabricated using a SWCNT electrode, ionogel dielectric, SnO or SnO_2_ semiconductor, and a gold top electrode. Electrochemical impedance spectroscopy (EIS) measurements on the MISM devices were also conducted using the VersaSTAT 4. Trace water content in the ionic liquids was quantified via Karl Fischer titration (Metrohm 852 Titrando).

### 4.4 SnO EGTs Fabrication

p‐Type SnO semiconductor films were grown and patterned using a previously reported ALD process [61, 62]. Source and drain electrodes were defined via photolithography, with a channel width (W) of 500 µm and a length (L) of 100 µm. Ni and Au were sequentially deposited onto the SnO by e‐beam and thermal evaporation, respectively. To suppress parasitic capacitance, an Al_2_O_3_ passivation layer was deposited over the electrodes by ALD. Top‐gated EGTs were completed by sequentially laminating the ionogel dielectric and SWCNT gate electrode onto the semiconductor. For balanced operation of logic gates, the channel dimensions of the n‐SnO_2_ transistors were adjusted to match the current level of the p‐SnO transistors, with the channel width and length set to 50 and 100 µm, respectively.

### 4.5 Electrical Characterization of EGTs and Logic Gates

All measurements were conducted under ambient conditions using a probe station (MS‐Tech) connected to a semiconductor parameter analyzer (Keithley 4200). The hole and electron mobilities in the saturation regime were extracted using the following equation:

(1) $$\mu_{sat}=\frac{2L}{W}\frac{s^{2}}{C_{i}},\left(S=\frac{\sqrt[{d}]{I_{D,sat}}}{dV_{G}}\right)$$

where *C_i_* is the specific capacitance extracted from EIS measurements using SWCNTs/ionogel/SnO or SnO_2_/gold capacitors at 100 Hz. The *C_i_* was determined to be 5.8 µF cm^−2^ for p‐SnO and 8.0 µF cm^−2^ for n‐SnO_2_. In addition, the effective channel capacitance was independently estimated from the gate‐current response using:

(2) $$I_{G}=C\frac{dV_{G}}{dt}=Cr_{V}$$

where d*V_G_*/*dt* corresponds to the gate‐voltage scan rate (*r_V_*) (Figure S13). This approach provides a direct measure of electrochemical charge accumulation in the channel. The capacitances extracted from the *I_G_*–*r_V_* plots were 6.4 µF cm^−2^ for p‐SnO and 12.6 µF cm^−2^ for n‐SnO_2_. The resulting carrier mobilities were 0.15 cm^2^ V^−1^ s^−1^ for p‐SnO and 0.74 cm^2^ V^−1^ s^−1^ for n‐SnO_2_. The area‐normalized charge density (*p*_A_) was calculated as follows: [63, 64]

(3) $$p_{A}=\frac{Q}{eA}=\frac{\int I_{G}dV_{G}}{r_{V}eA}$$

where *r*_V_ represents the constant gate voltage scan rate (*r*_V_ = d*V*_G_/d*t*, fixed at 15 mV s^−1^ for p‐SnO and 40 mV s^−1^ for n‐SnO_2_). The authors used ChatGPT to improve language and readability. After using ChatGPT, the authors carefully reviewed and edited the manuscript as necessary and took full responsibility for the content of the publication.

## Author Contributions

**Keun Hyung Lee**: conceptualization, supervision, funding acquisition, Writing – review and editing, validation. **Taek‐Mo Chung**: investigation. **Dong Hyun Park**: data curation, investigation, writing – original draft, formal analysis. **Seong Keun Kim**: investigation, supervision, writing – review and editing. **Min Su Kim**: validation, investigation. **In‐Hwan Baek**: conceptualization, supervision, funding acquisition, writing – review and editing. **Seung Ho Ryu**: investigation, formal analysis, data curation.

## Conflicts of Interest

The authors declare no conflicts of interest.

## Supporting information




**Supporting File**: advs76809‐sup‐0001‐SuppMat.docx.

## Data Availability

Data pertaining to this work can be obtained from the authors upon request.
