# Supplementary material for: Selective Polarity Control of Metal Oxide Semiconductors for Complementary Logic Gates
Source: Adv Sci (Weinh). 2026 Jul 29:e76809. Online ahead of print. doi: 10.1002/advs.76809 (PMC13418052; doi:10.1002/advs.76809)
Supplement: Supplementary file 1 — Supporting File: advs76809‐sup‐0001‐SuppMat.docx. [file ADVS-9999-e76809-s001.docx]

Supporting Information

**Selective Polarity Control of Metal Oxide Semiconductors for Complementary Logic Gates**

*Dong Hyun Park, Seung Ho Ryu, Min Su Kim, Taek-Mo Chung, Seong Keun Kim*,*

*In-Hwan Baek*, and Keun Hyung Lee**

D. H. Park, M. S. Kim, I.-H. Baek, and K. H. Lee

Department of Chemistry and Chemical Engineering, Education and Research Center for Smart Energy and Materials

Inha University, Incheon, 22212, Republic of Korea

E-mail: [kh.lee@inha.ac.kr](mailto:kh.lee@inha.ac.kr), baek@inha.ac.kr

S. H. Ryu, S. K. Kim
Electronic and Hybrid Materials Research Center

Korea Institute of Science and Technology, Seoul 02792, Republic of Korea

E-mail: [s.k.kim@kist.re.kr](mailto:s.k.kim@kist.re.kr)

S. H. Ryu, S. K. Kim

KU-KIST Graduate School of Converging Science and Technology

Korea University, Seoul 02841, Republic of Korea

T.-M. Chung

Division of Advanced Materials

Korea Research Institute of Chemical Technology, Daejeon 34114, Republic of Korea

Keywords: complementary logic, electrochemical doping, electrolyte-gated transistor, metal oxide semiconductor, polarity conversion

**Table S1.** Device characteristics of the p-type SnO and n-type SnO_2_ EGTs.

| Polarity | V_th_  [V] | ON/OFF current ratio | *C_i_^a^*  [*μ*F/cm^2^] | *C_i_^b^*  [*μ*F/cm^2^] | *μ^a^*  [cm^2^/V·s] | *μ^b^*  [cm^2^/V·s] | Subthreshold  swing (SS)  [mV/dec] | Charge  density  [cm^−2^] |
| --- | --- | --- | --- | --- | --- | --- | --- | --- |
| p (SnO) | 0.27  ± 0.02 | (1.38 ± 0.32)  × 10^4^ | 5.8  ±0.01 | 6.4  ±0.24 | 0.17  ±0.01 | 0.15  ±0.01 | 163  ± 10 | (0.33 ± 0.18)  × 10^14^ |
| n (SnO_2_) | −0.14  ± 0.07 | (1.03 ± 0.25)  × 10^3^ | 8.0  ±0.05 | 12.6  ±0.51 | 1.17  ±0.12 | 0.74  ±0.03 | 277  ± 14 | (0.54 ± 0.28)  × 10^14^ |

*C_i_^a^ and μ^a^ were extracted from EIS measurements of a MISM device.*

*C_i_^b^ and μ^b^ were extracted from the slope of the I_G_ vs. r_V_ curves in Figure S13.*

**Table S2. Comparison of reported polarity-control strategies in semiconductor devices.**

| **Ref.** | | **Material** | **Polarity control mechanism** | | | **In-situ polarity conversion** | **Operating Voltage (V)** |
| --- | --- | --- | --- | --- | --- | --- | --- |
| **This work** | | **Single SnO-derived SnO/SnO_2_** | **Electrochemical oxidation** | | | **O** | **1.5** |
| ^1^ | WSe₂ + Cl-SnSe_2_ vdW contact | | | Contact engineering | X | | ±6 |
| ^2^ | MoTe_2_, WSe_2_ | | | Reversible photodoping | O | | ±20 |
| ^3^ | BP homojunction | | | Electrostatic polarity control | O | | ±1 |
| ^5^ | MoS_2_ + vdW metal contacts | | | Work-function tuning | X | | ±60 |
| ^6^ | PdSe_2_ | | | Metal contact engineering | X | | ±20 |
| ^7^ | MoTe_2_ | | | O_2_ adsorption/desorption by air/vacuum annealing | O | | ±5 |
| ^8^ | WSe_2_ + WOx | | | Surface acceptor formation | X | | ±40 |
| ^9^ | MoTe_2_ | | | Al_2_O_3_-induced electron transfer doping | X | | ±5 |
| ^44^ | hBN/MoTe_2_ | | | Schottky contact engineering by thermal annealing | O | | ±5 |
| ^45^ | MoTe_2_ | | | O_2_ adsorption/desorption | O | | ±5 |
| ^46^ | MoTe_2_ + PMMA/e-beam | | | O_2_/H_2_O adsorption control | O | | ±5 |
| ^47^ | hBN/ReSe_2_/hBN | | | Photo-charge trapping | O | | ±80 |

**Table S3. Comparison of the electrical performance of reported oxide-semiconductor-based EGTs.**

| **Ref.** | **Channel** | **Electrolyte / Gate dielectric** | **Polarity** | **μ**  **[cm^2^/Vs]** | **ON/OFF**  **current ratio** | **SS**  **[V/dec]** |
| --- | --- | --- | --- | --- | --- | --- |
| **This work** | **p-SnO** | **PVDF-HFP/[EMI]**  **[TFSI] ionogel** | **p-type EGT** | **0.17** | **1.4×10^4^** | **0.16** |
|  | **n-SnO_2_ (converted from SnO)** | **PVDF-HFP/[EMI]**  **[TFSI] ionogel** | **n-type EGT** | **1.17** | **~10^3^** | **0.28** |
| ^48^ | Cu_2_O | PVA/PC/LiClO_4_ | p-type EGT | 0.15 | 10^2^ | 0.70 |
| ^49^ | Cu_2_O | PVA/PC/LiClO_4_ | p-type EGT | - | ~10^3^ | - |
| ^50^ | SnO_2_ | [EMIM][TFSI] ionic liquid | n-type EGT | 0.16 | ~10^4^ | – |
| ^51^ | SnO_2_ | PBS electrolyte | n-type EGT | 1.6 | ~10^3^ | 0.09 |
| ^52^ | Mg-doped SnO_2_ | PEO:LiClO_4_ | n-type EGT | 1.2 | ~10^4^ | 0.80 |
| ^53^ | ZnO | SEAS/[EMI][TFSI] ionogel | n-type EGT | 2.0 | ~10⁵ | – |
| ^54^ | ITO | Sodium alginate electrolyte | n-type EGT | 9.8 | 10^3^–10^4^ | 0.20 |
| ^55^ | IZO | PVDF-HFP/[EMI][TFSI] ionogel | n-type EGT | 41.66 | 6.8×10^4^ | 0.27 |
| ^56^ | In_2_O_3_/Er_2_O_3_ | PEO:LiClO_4_ | n-type EGT | – | – | – |
| ^57^ | IGZO | PBS electrolyte | n-type EGT | – | 1.4×10^3^ | 0.19 |
| ^58^ | IGZO | PBS electrolyte | n-type EGT | – | ~10^6^ | 0.08 |
| ^59^ | IGZO | AAm-TFEA/[EMIM][TFSI] ionogel | n-type EGT | 4.08 | ~10^5^ | – |
| ^60^ | IGZO | NCM ionogel | n-type EGT | – | 6.6×10^6^ | 0.06 |

**Table S4.** Trace water concentrations in [EMI][TFSI], [EMI][BF_4_], and [BTMA][TFSI].

|  | wt% | mol% |
| --- | --- | --- |
| [EMI][TFSI] | 0.067 | 1.44 |
| [EMI][BF_4_] | 0.045 | 0.49 |
| [BTMA][TFSI] | 0.038 | 0.82 |

**
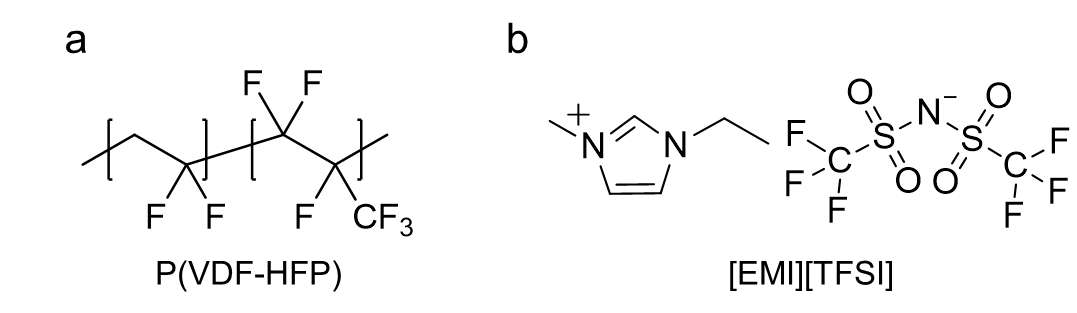
**

**Figure S1.** Chemical structures of a) the polymer matrix P(VDF-HFP), and b) the ionic liquid ([EMI][TFSI]) comprising the ionogel.

**
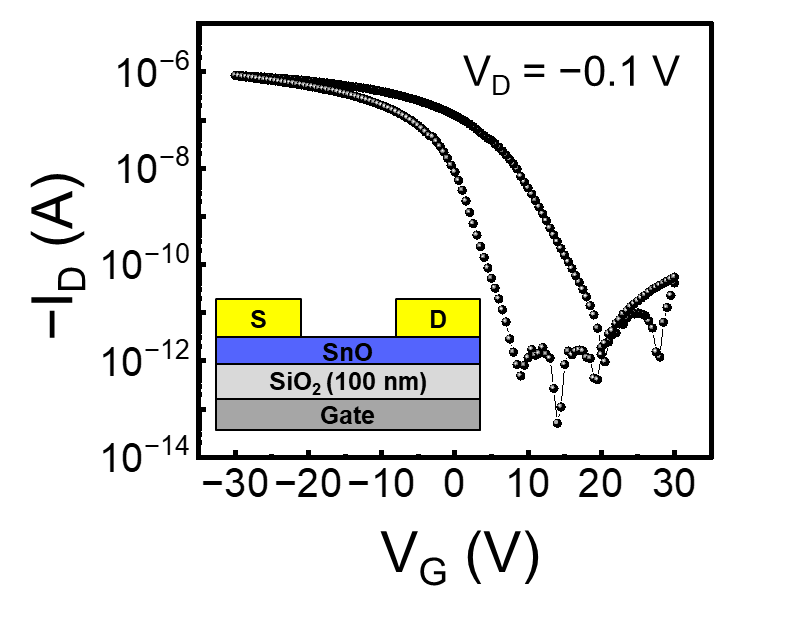
**

**Figure S2.** *I*_D_*−V*_G_ transfer curve of a SnO semiconductor gated with a SiO_2_ dielectric, measured at *V*_D_ = −0.1 V. The device configuration is shown in the inset.

**
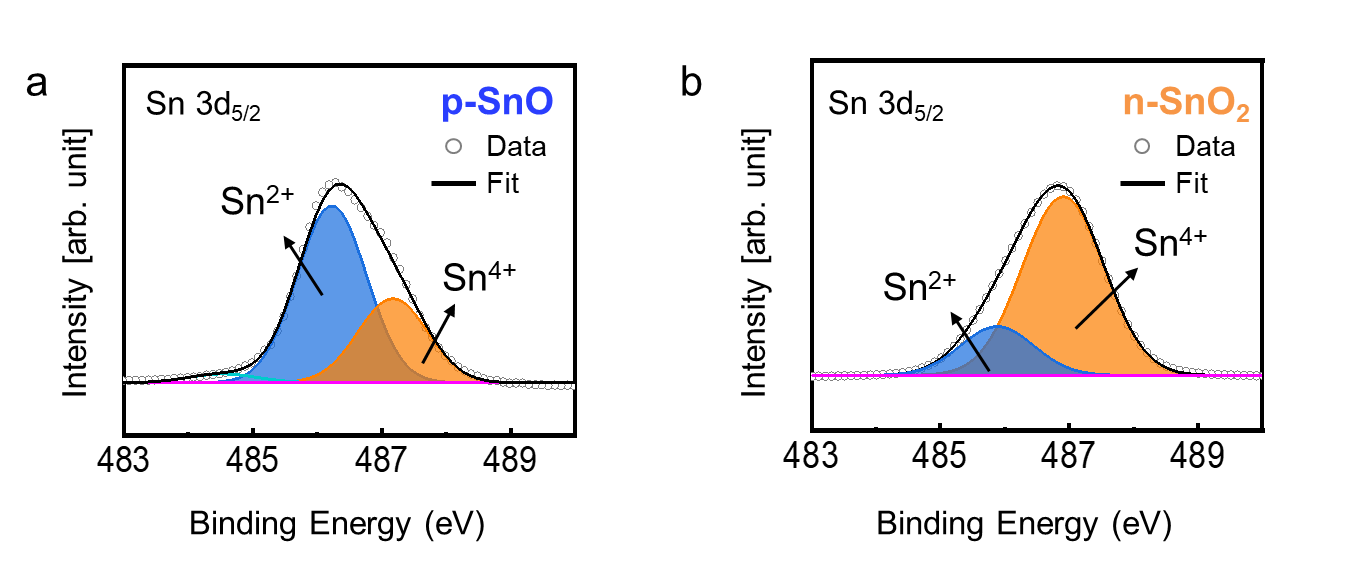
**

**Figure S3.** XPS analysis of Sn oxidation states. XPS spectra of Sn 3d_5/2_ for a) pristine p-SnO and b) n-SnO_2_. The binding energies of Sn^2+^ and Sn^4+^ are located at 486.1 eV and 487.0 eV, respectively.

**
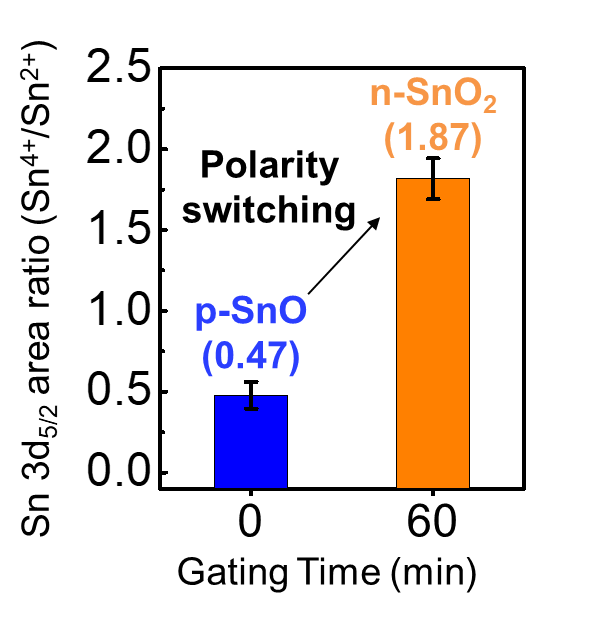
**

**Figure S4.** Peak area ratio of Sn^4+^/Sn^2+^ from the Sn 3d_5/2_ before (p-SnO) and after polarity switching (n-SnO_2_).

**
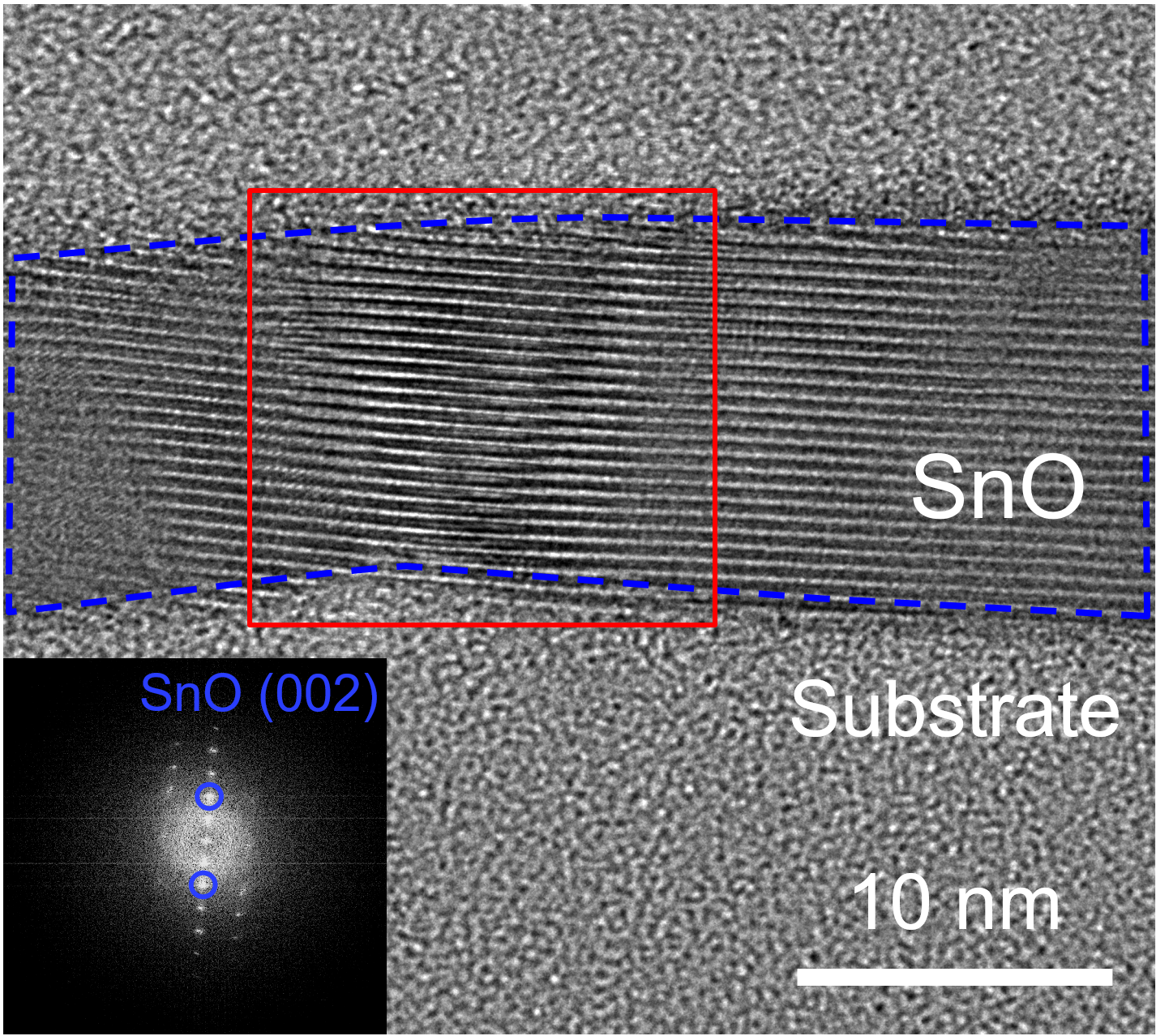
**

**Figure S5.** Cross-sectional HR-TEM image of the SnO thin-film and its corresponding FFT of the red-boxed region.


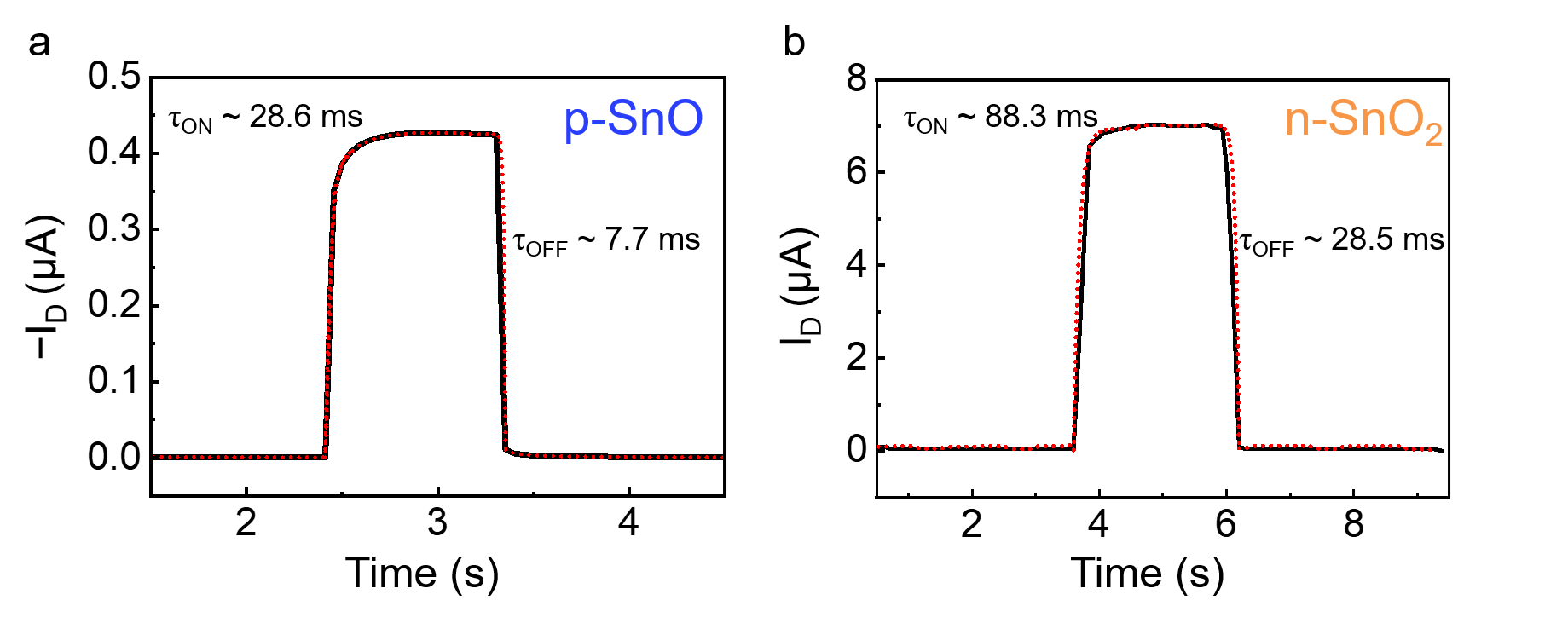


**Figure S6.** Temporal response of p-SnO and converted n-SnO_2_ transistors under square gate-voltage pulses. The gate voltage was switched between 0.6 and −0.2 V for p-SnO and between 0 and 0.7 V for n-SnO_2_. Red dotted lines indicate single-exponential fits used to extract the turn-on (τ_ON_) and turn-off (τ_OFF_) time constants.

*
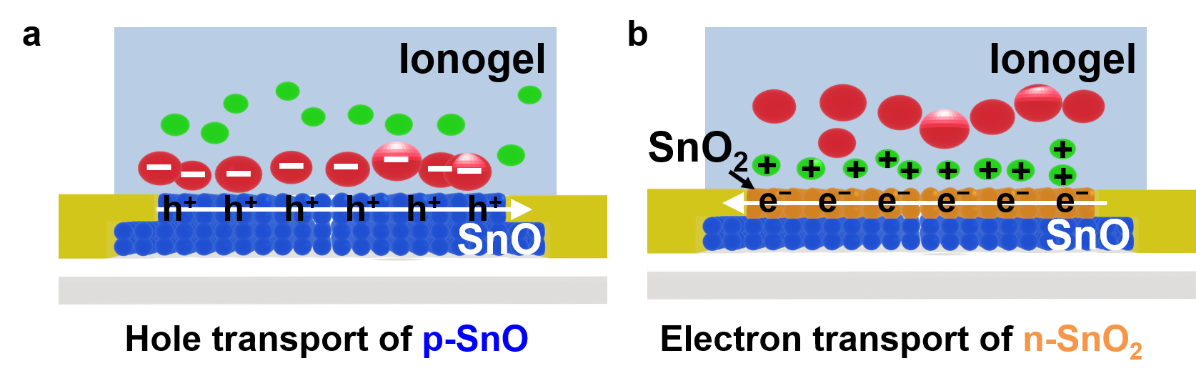
*

**Figure S7.** Schematic illustrations of the expected conduction pathways in (a) pristine p-SnO and (b) electrochemically converted n-SnO_2_ EGTs.


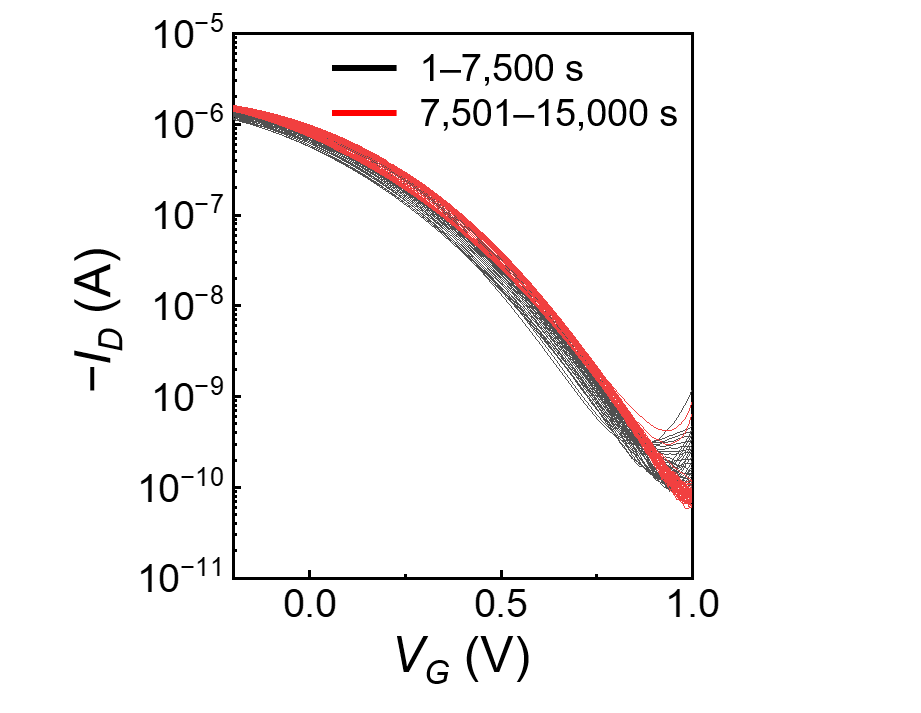


**Figure S8.** Transfer curves of the p-SnO EGT measured during continuous operation for 15000 s. Black and red curves correspond to measurements recorded during 1–7500 s and 7501–15000 s, respectively.

**
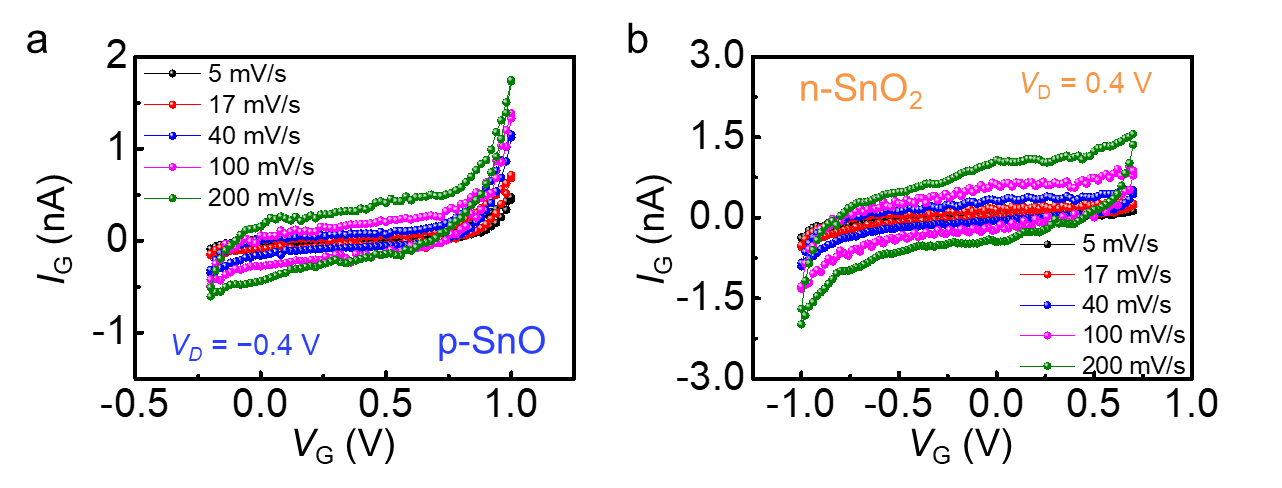
**

**Figure S9.** Gate current characteristics of EGTs at varying scan rates. *I*_G_*−V*_G_ curves measured at scan rates of 5, 17, 40, 100, and 200 mV s^−1^ for a) p-SnO and b) n-SnO_2_ EGTs. Measurements were performed at *V*_D_ = −0.4 V for the p-type EGT and 0.4 V for the n-type EGT.

**
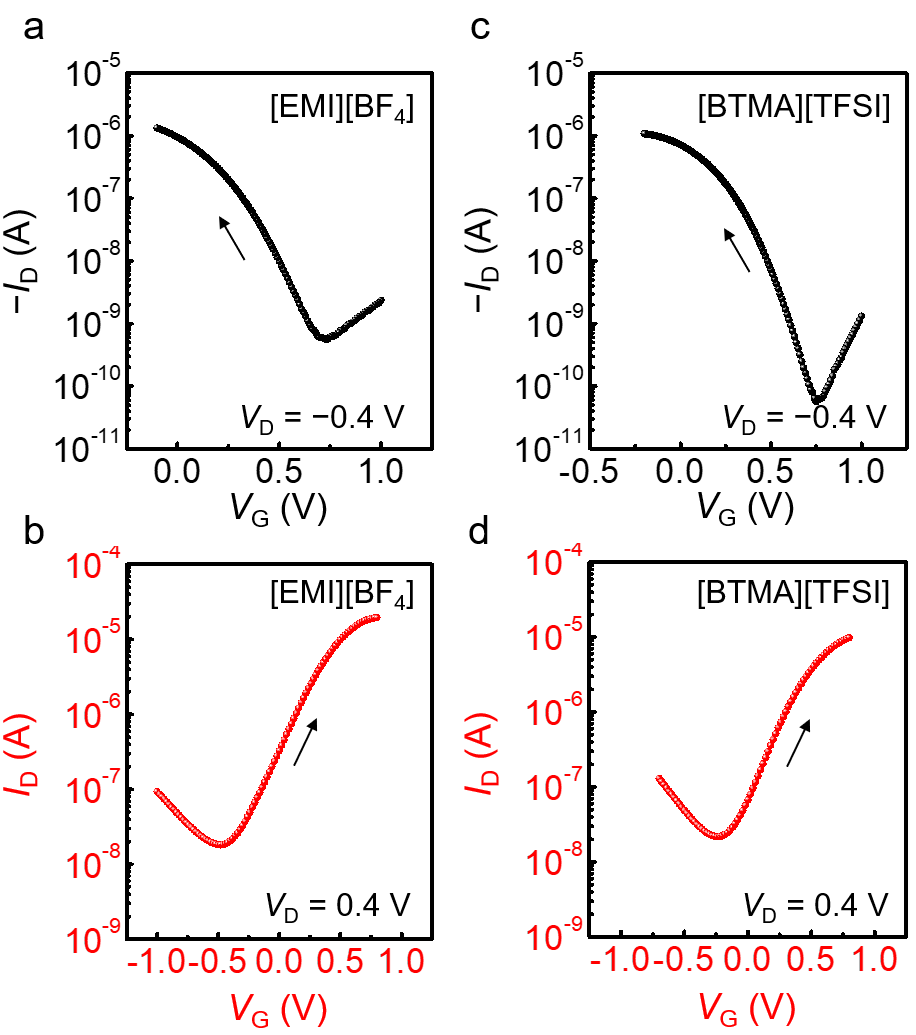
**

**Figure S10.** Electrical characteristics of SnO EGTs gated with different electrolytes. Transfer curves of p-type EGTs gated with a) [EMI][BF_4_] and c) [BTMA][TFSI] ionogels, and their corresponding n-type EGTs after polarity switching in b) and d), respectively. *V*_D_ = −0.4 V and 0.4 V for p- and n-type EGTs, respectively.


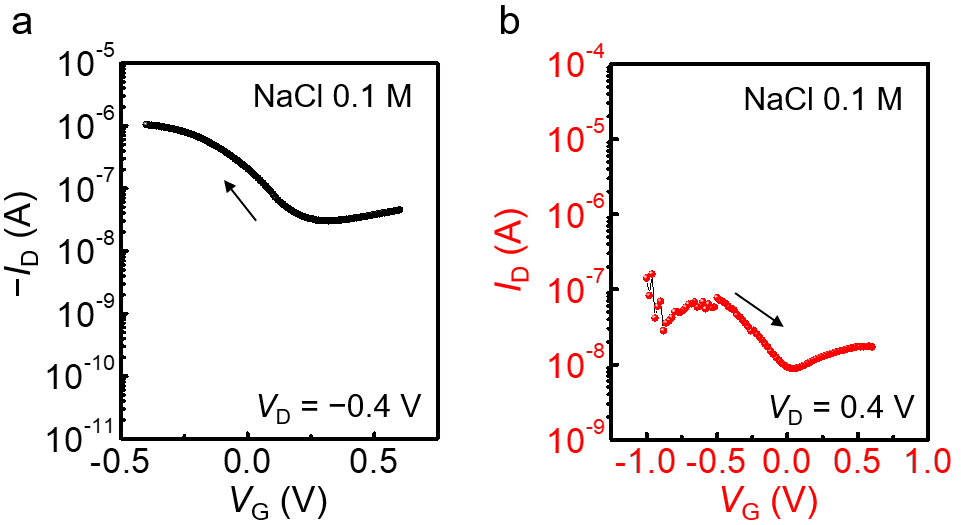


**Figure S11.** Electrical characteristics of SnO gated with aqueous electrolyte. Transfer curves of a) p-type and b) n-type operation gated with 0.1 M NaCl aqueous electrolyte under ambient conditions.

**
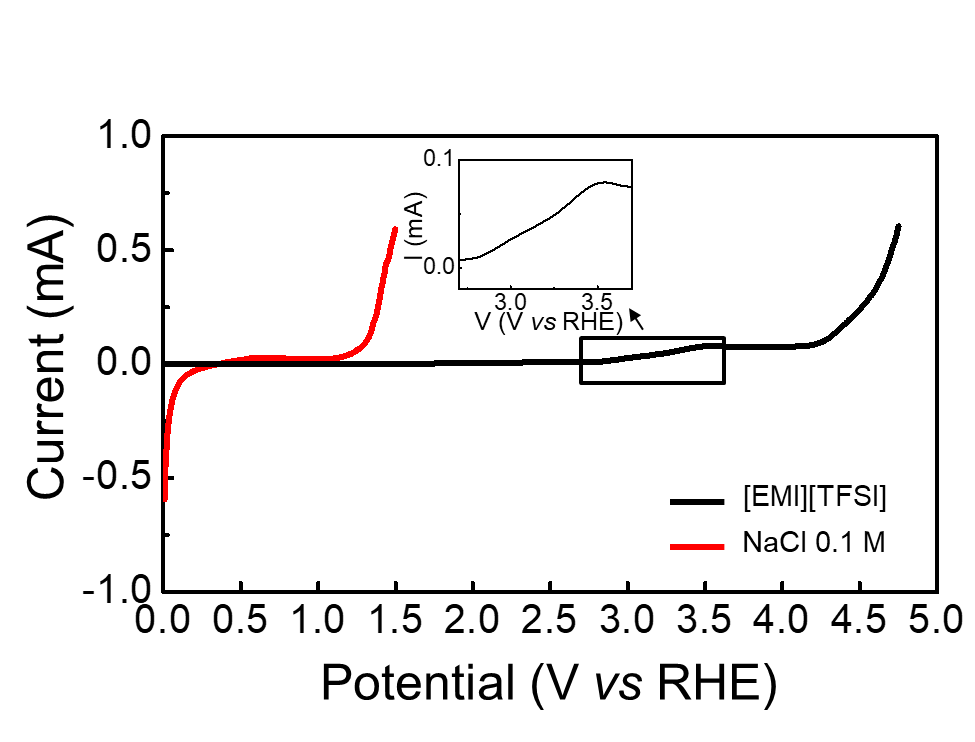
**

**Figure S12.** Linear sweep voltammetry (LSV) curves for the [EMI][TFSI] and 0.1 M NaCl electrolytes. The measurements were performed using a SUS/electrolyte/Pt cell with an AgNO_3_ reference electrode. All potentials are referred to the reversible hydrogen electrode (RHE).


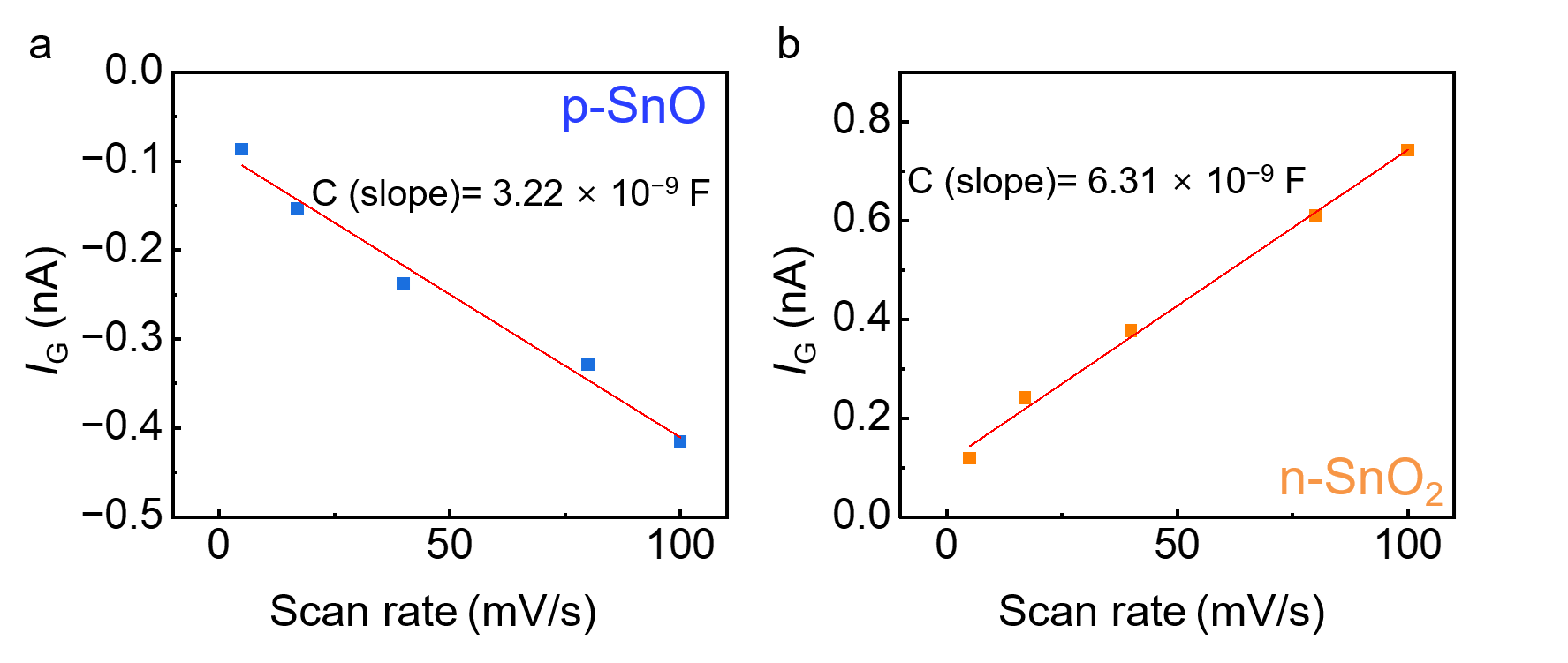


**Figure S13.** Determination of areal capacitance from the gate-current response. Gate current (*I_G_*) as a function of gate-voltage scan rate (*r_V_*) for (a) p-SnO and (b) n-SnO_2_ EGTs. The capacitance obtained from the slope of the linear fit was normalized by the channel area to yield the areal capacitance.
